# Opportunities for Laboratory Testing to Inform Antimicrobial Use for Bovine Respiratory Disease: Application of Information Quality Value Stream Maps in Commercial Feedlots

**DOI:** 10.3390/antibiotics13090903

**Published:** 2024-09-21

**Authors:** Simon J. G. Otto, Colleen M. Pollock, Jo-Anne Relf-Eckstein, Lianne McLeod, Cheryl L. Waldner

**Affiliations:** 1 HEAT-AMR (Human-Environment-Animal Transdisciplinary Antimicrobial Resistance) Research Group, School of Public Health, University of Alberta, Edmonton, AB T6G 1C9, Canada; colleen.pollock@usask.ca (C.M.P.); jer128@mail.usask.ca (J.-A.R.-E.); 2 Healthy Environments Thematic Area Lead, School of Public Health, University of Alberta, Edmonton, AB T6G 1C9, Canada; 3 Department of Large Animal Clinical Sciences, Western College of Veterinary Medicine, University of Saskatchewan, Saskatoon, SK S7N 5B4, Canada; lianne.mcleod@usask.ca

**Keywords:** feedlot calves, value stream map, bovine respiratory disease, laboratory testing, antimicrobial resistance, antimicrobial stewardship

## Abstract

Background/Objectives: The implementation of information quality value stream maps (IQ-VSMs) in food animal production systems can increase our understanding of the opportunities and challenges when using laboratory testing for antimicrobial resistance (AMR) to support antimicrobial stewardship (AMS). Our objectives were to (1) explore the implementation of information quality value stream mapping as a continuous improvement tool to inform decisions for bovine respiratory disease (BRD) management and AMS and (2) apply the information quality dimensions to identified Kaizen opportunities for the integration of laboratory data into BRD management systems to assess the appropriateness of BRD treatment plans in western Canadian feedlot production. Methods: A ‘Current State’ IQ-VSM outlined the processes, available information, information processing steps, and control decisions contributing to BRD management and treatment in commercial western Canadian feedlots, recognizing that laboratory BRD pathogens and AMR data are typically not part of BRD management. Results: The ‘Future State’ IQ-VSM incorporated Kaizen opportunities for improvement, including (i) the strategic collection of respiratory samples from representative samples of calves for laboratory analysis, regardless of clinical BRD status, (ii) compilation of laboratory data at the pen and feedlot levels, and (iii) analysis of pen- and feedlot-level laboratory data to inform the veterinarian’s assessment of the appropriateness of current BRD treatment plans. Conclusions: The IQ-VSMs provided a valuable framework to visualize the integration of BRD pathogen and AMR laboratory data to support AMS and address any potential future testing requirements.

## 1. Introduction

Laboratory testing for antimicrobial resistance (AMR) can contribute to antimicrobial stewardship (AMS) in food animal production by supporting diagnostic and treatment decisions [1]. In 2015, the World Health Organization Global Action Plan for AMR [2], in conjunction with the Food and Agriculture Organization and the World Organisation for Animal Health, highlighted development and investment in laboratory diagnostic tests for AMR detection as a key objective to support global AMS [1]. Food animal production operations are complex biological systems where appropriate antimicrobial use (AMU) is necessary to protect animal health and welfare [3]. The integration of laboratory testing to detect pathogens and AMR and inform AMU remains challenging due to delays from sample collection to results, costs, and limitations and challenges in the interpretation of current phenotypic and molecular tests [4]. 

Value stream maps (VSMs) are used to visualize, analyze, design, and continuously improve factory-based production by identifying waste (losses), reducing process cycle times, and implementing process improvement in alignment with lean manufacturing principles [5]. Mapping allows for the identification of ‘Kaizen’, which are continuous improvement opportunities within the system. Jones and Womack [6] advanced the original visual mapping techniques for plant-level processes and extended the tool to map entire supply chains. Today, value stream mapping recognizes the digitization and interconnectivity of systems as part of the Industrial Revolution 4.0 (IR4.0) [7]. Busert and Fay [8] first identified the importance of information quality in the decision-making process for VSMs and proposed the ‘Information Quality-VSM’ (IQ-VSM) thereafter [9,10,11]. The IQ-VSM accounts for production processes, information, information processing, and control decisions in a production system and considers three important aspects of information quality: accuracy, frequency, and granularity [9,10,11].

Bovine respiratory disease (BRD) is the leading cause of morbidity and mortality in North American feedlot cattle [12,13]. It represents the most common reason for injectable AMU in western Canadian feedlot cattle, including macrolide and tetracycline antimicrobials [14]. As a multifactorial disease complex associated with numerous bacteria and viruses [15,16], laboratory testing would ideally inform clinical management and antimicrobial treatment decisions [12,15,16]. However, current BRD treatment protocols in feedlot production usually rely on clinical signs and known risk factors. These protocols typically do not include laboratory testing of individual animals to inform AMU due to the importance of timely treatment for a successful outcome [17] and the expected delays related to obtaining test results from the laboratory [12].

While testing each individual calf before treatment is not currently practical in large commercial feedlots, there is an opportunity to use laboratory data from their pen mates or contemporary management cohort to inform BRD antimicrobial treatment protocols. Limited research to date has explored the potential for pen-level ante mortem sampling and laboratory testing strategies to inform AMU protocols for BRD treatment in feedlot calves. The IQ-VSM provides a framework for identifying opportunities to integrate laboratory testing into the information management systems currently used by veterinarians to develop and update AMU protocols for BRD management in western Canadian feedlots. Cattle entering western Canadian feedlots are currently assigned to a BRD risk category upon entry to the feedlot, which then guides the decision on an antimicrobial protocol for BRD metaphylaxis [14]. These decisions are made within seconds within the animal handling system, meaning that laboratory testing to inform them is not feasible with current technology. The AMS opportunity lies in the potential use of laboratory testing at the animal group level to assess BRD treatment protocols for subsequent animals that get sick in terms of identifying circulating bacterial BRD pathogen and phenotypic AMR or AMR gene (ARG) complements.

Value stream mapping has been applied to food processing systems and food service industries [18,19,20]. However, it has not been applied to food animal production systems, particularly to understand how laboratory testing for bacterial pathogens and AMR/ARGs could be integrated to continuously improve the management of animal diseases and promote AMS. The overall aim of this study was to investigate information quality value stream mapping as a tool to identify how and where laboratory testing strategies can be implemented to support AMS in livestock production. The specific objectives were to (1) explore the implementation of information quality value stream mapping as a continuous improvement tool to inform decisions for BRD management and AMS in western Canadian feedlots and (2) apply the information quality dimensions to identified Kaizen opportunities for the integration of laboratory data into BRD management systems to assess the appropriateness of BRD treatment plans in western Canadian feedlot production. The resulting IQ-VSMs provided a detailed map of BRD management information flows in western Canadian commercial feedlot production. The comprehensive depiction of the ‘Current’ and ‘Future State’ IQ-VSMs demonstrated the potential for the integration of laboratory testing for BRD pathogens and AMR to support animal health and AMS.

## 2. Results

### 2.1. Overview and Environmental Context: IQ-VSM for BRD Treatment Plans in Commercial Feedlots

Most beef calves enter the western Canadian feedlot system in the fall. These tend to be recently weaned calves between 450 and 600 lbs, sourced from auction markets and considered high risk for BRD. Once in the feedlot, these calves will typically spend 200 days or more in pens of up to 200–300 animals on an increasingly concentrate-based diet for growth and finishing. Western Canada feeds most Canadian beef calves and contains the largest number and size of feedlots in the country [21]. For example, as of January 2023, Canfax reported capacity to feed 1.7 million cattle in 171 feedlots in the western provinces of Alberta and Saskatchewan [22]. 

The peak risk period for BRD is the first few weeks after feedlot entry. High-risk calves are typically administered an antimicrobial for BRD metaphylaxis at feedlot arrival, with additional injectable antimicrobial treatment for BRD only if they are identified by pen riders as displaying clinical signs [14]. The BRD management and treatment protocols for use of antimicrobials by feedlot staff are usually designed by consulting feedlot veterinarians, and all medically important antimicrobials in Canada are available only by veterinary prescription. 

We created a Current-State IQ-VSM to outline data collection and processing steps relevant to BRD management and treatment in moderate to large commercial western Canadian feedlots. We then created a Future-State IQ-VSM to identify Kaizen that represent opportunities to (i) collect respiratory samples for laboratory analysis for BRD pathogen and AMR detection, (ii) compile individual calf laboratory data at the pen and feedlot levels, and (iii) analyze pen- and feedlot-level laboratory data to inform the veterinarian’s assessment of the appropriateness of BRD treatment plans.

### 2.2. Current State IQ-VSM for BRD Treatment Plans in Commercial Feedlots

#### 2.2.1. Process Lane (Bottom)

**Figure 1:**
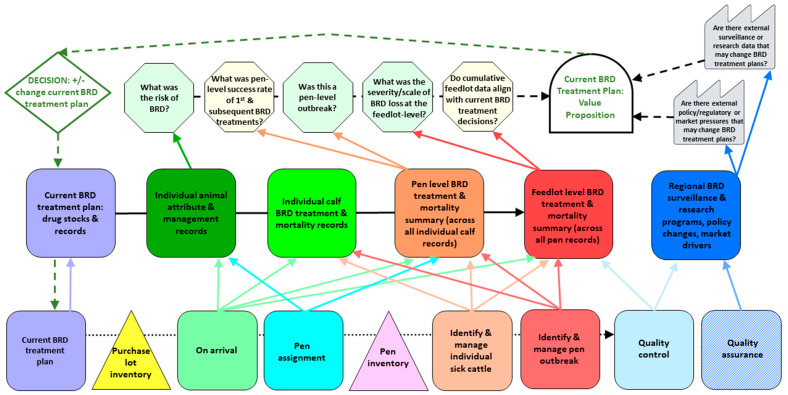
The simplified Current State IQ-VSM (Table 1, Table 2 and Table 3) for BRD treatment plans used in a western Canadian commercial beef cattle feedlot production system. The bottom row is the Process Lane (Table 1), the middle row is the Information Lane (Table 2), and the top row is the Information Processing Lane (Table 3). The detailed Current State IQ-VSM is provided in Supplementary Figure S2. Dotted horizontal arrow: associated information (static, order-independent data). Solid arrows: processed/to be processed information (dynamic, order-dependent data updated in real-time). Dashed black arrow: information processing related to the control decision. Dashed green arrow: control decision feedback loop.

The information resources for each production process in a feedlot are represented by a series of horizontal process boxes with associated vertical data source boxes, along with inventory triangles (Figure 1, Supplementary Figures S1 and S2). The components of each production process and inventory of the IQ-VSM for the BRD treatment plan are detailed in Table 1. The components were initially identified by leveraging the experience of team members and then refined through iterative discussion. The resources depicted in the Process Lane incorporate the three groups of IR4.0 information logistics proposed by Meudt et al. [23], namely data generation and transfer, data processing and storage, and data utilization.

This Process Lane comprises static, retrospective information and processes for collecting data that govern feedlot operations for managing BRD. It is information that arrives with the calves as they enter the feedlot but does not change in real time as BRD develops and transmission of pathogens and AMR proceeds. The Process Lane represents the ‘recipes’ of existing types of data about and related to BRD that are collected by the proprietary and confidential feedlot management software programs that feedlot veterinarians use to link to and collect data from feedlots under their care. All information flows horizontally in this lane and is static, in that it is based on retrospective data that do not change with real-time BRD incidence. Note that each production process is labeled with a Data Type number to cross-reference to the middle Information Lane (Figure 1, Supplementary Figure S2).

**Table 1:** Process Lane: Processes, inventory, information resources (sources of the information for the processes), and types of data (types of static information that inform the processes) for the Current State IQ-VSM (Figure 1, Supplementary Figure S2) contributing to BRD treatment plans that might be used in a western Canadian commercial beef cattle feedlot production system.

Production Processes	Information Resources	Types of Static Data
Current BRD treatment plan	In-house proprietary protocols and algorithms: Veterinary clinic proprietary research metadata with restricted access Feedlot proprietary metadata with restricted access that links to and makes up part of the veterinary clinic data Government policies and regulations Customer preferences	Data Type 1 Choices of therapeutic drugs (list of options within BRD treatment protocol for first and subsequent treatments) Dosage regime Suspect causal BRD pathogens Historical in-house data (e.g., disease investigations, laboratory results, post-mortems, research projects, etc.) External data: ○ Government and/or industry policies and regulations influencing AMU ○ Market-driven policies influencing AMU Alternative BRD treatment protocols
Material inventory 1: incoming purchase lot animal inventory	Purchase lot records	Data Type 2—purchase lot records to capture: Record of origin of purchase lot and associated information (e.g., auction vs. direct source) Lot attributes: number of animals, sex, breed/colour, weight, age category Vaccination history—if available
On-arrival process	In-house proprietary protocols and algorithms: Assessment of BRD risk category Animal processing In-house proprietary databases for electronic data capture using weigh scales, tablets, scanners and sensors linked to RFID tag Upload captured data to feedlot management software	Data Type 3—calf records to capture: RFID, visual tag, weight, sex, pregnancy status, castration, dehorned, parasite treatment, other vaccinations BRD risk category assignment and antimicrobial metaphylaxis options Antimicrobial metaphylaxis used and vaccination for BRD
Pen assignment process	In-house proprietary protocols and algorithms: Assignment of calves to pens and associated information from purchase lot and on-arrival processing Data capture and upload to feedlot management software	Data Type 4—pen records and tools to summarize: (Record of pen identification and membership) Assign pen BRD risk category Numbers of animals allocated to pens Pen summary of incoming animal attributes (average weight, sex, health status, metaphylaxis)
Material inventory 2: pen animal inventory	Pen records within feedlot management software	Data Type 5—tools to summarize: Counts of animals per lot and pen Arrival dates of animals organized by lots and by pen
Process to identify and manage individual sick animals	In-house proprietary protocols and algorithms: Pen-checking (based on BRD risk category, season) to identify calves with BRD BRD diagnosis Pen-checker experience/training BRD treatment data capture and upload to feedlot management software	Data Type 6—record system and tools to summarize: Subjective observational data about change in cattle behaviour that could indicate BRD (e.g., depression, isolation, off feed) Objective clinical data (e.g., rectal temperature, respiratory rate/quality, other indicators of BRD) Record of BRD diagnosis Record of previous BRD treatment Record of BRD current treatment
Process to identify and manage a pen outbreak	In-house proprietary protocols and algorithms: Process for identifying a pen outbreak based on user-defined BRD treatment threshold and/or BRD mortality threshold Action when a BRD outbreak has been identified Data capture and upload to feedlot management software	Data Type 7—process to collect and summarize: Counts of animals treated once for BRD Counts of repeated treatment(s) for BRD (e.g., treatment failure) Pen-level feed consumption Counts of animals sent to the chronic pen Counts of animals dead due to BRD Gross post mortem and other diagnostics Outbreak pen antimicrobial treatment and/or re-vaccination
Quality control process	In-house proprietary protocols and algorithms: Veterinary clinic BRD analysis and decision-support tools Post-mortem images and clinical videos Other diagnostics Data capture and upload to feedlot management software	Data Type 8—process to collect and summarize: Counts of BRD (individual, pen incidence) Counts of BRD treatment failure/repeat treatments Counts of animals sent to the chronic pen Counts of BRD mortalities Collated post-mortem and diagnostic results
Quality assurance process	Shared resources (in-house +/− external): Regional surveillance Research activities Policies and regulations	Data Type 9: CIPARS: AMR and AMU surveillance program BRD and related research studies Market studies

The first production process is the current BRD treatment plan (Table 1, Figure 1, Supplementary Figure S2), which includes the collection of BRD treatment protocols based on feedlot and veterinary clinic clinical and research metadata, the requirements of government policies and regulations, and requirements for specific marketing chains. The first inventory box relates to the purchase lot records accompanying the calves at feedlot arrival. The next on-arrival production process includes all the in-house protocols and algorithms associated with on-arrival processing, capturing of incoming inventory data, BRD risk categorization, events that happen during on-arrival processing, and data capture to feedlot management software. The next production process, pen assignment, includes animal allocations based on BRD risk, weight, and sex, and pen-level summaries of animal attributes captured into feedlot management software, which then links to the pen inventory. 

After pen assignment, there are two production processes to identify and manage individual sick cattle and then pen outbreaks (Table 1, Figure 1, Supplementary Figure S2). This is followed by the important quality control process step: feedlot analysis of BRD occurrence and decision-support tools, data capture for field post-mortems, and overall capture by the feedlot records management system. The data include the system to summarize and review counts of BRD incident cases, treatment successes/failures/repeats, assignments to the chronic pen, mortality counts, and collated post-mortem +/− additional diagnostic results. The final process is quality assurance that is linked to shared external resources such as regional surveillance for AMR and/or AMU (e.g., the Canadian Feedlot AMU/AMR Surveillance Program—CFAASP, within the Canadian Integrated Program for AMR Surveillance—CIPARS), any BRD and related research, market data or studies, and overarching policies. Surveillance data could be the counts of BRD pathogens isolated and the proportion that are resistant to the antimicrobials tested, as well as AMU data at the regional level (total mg of active ingredient distributed/sold for use in cattle (if available), or some other standardized AMU metric).

#### 2.2.2. Information Lane (Middle)

The BRD information flows in a western Canadian feedlot were mapped from the production processes in the Process Lane to the information processes in the middle Information Lane (Figure 1, Supplementary Figures S1 and S2), with the difference being the latter data change with time. The components of information flows and data for the BRD treatment plan pertaining to the middle Information Lane are detailed in Table 2. These represent the real-time BRD incidence, treatment, and associated data from the feedlot. 

**Table 2:** Information Lane: Real-time data and associated processes from the Current State IQ-VSM (Figure 1, Supplementary Figure S2) for BRD treatment plans that might be used in a western Canadian commercial beef cattle feedlot production system. Data Type is indicated in reference to the Data Types in Table 1, Figure 1, and Supplementary Figure S2.

Information Processes	Static Information ^1^	Dynamic Information
Current BRD treatment plan: drug stocks and records	Data Type 1	Drug inventory/supply: Drugs currently on hand for use Cost of drugs Ease of use of drug Drug access (supply chain availability)
Individual animal attribute and management records	Data Types 3 + 4	Purchase history: Purchase lot and age category on arrival Individual animal on-arrival processing: RFID tag and feedlot tag, sex, breed or colour, weight Vaccination on arrival BRD risk category and antimicrobial metaphylaxis on arrival Other processing checks/treatments on arrival (e.g., parasite treatment, castration, implants, pregnancy checking +/− aborting, dehorning) Pen assignment Record updates at re-processing +/− re-sorting
Individual calf BRD treatment and mortality records	Data Types 3 + 6 + 7	BRD risk assessment on arrival and BRD treatment protocol assignment Record of 1st BRD diagnosis and treatment Record of 2nd BRD diagnosis and treatment Record of 3rd BRD diagnosis and treatment Days on feed (DOF) when treating Assignment to chronic pen Mortality due to BRD Calf report: post-mortem and other diagnostic tests
Pen-level BRD treatment and mortality summary (across all individual calf records)	Data Types 3, 4, 6, 7	Pen-level summary of individual calf records for: Count and % of animals treated once for BRD Count and % of animals treated twice for BRD Count and % of animals treated three times for BRD Count and % of animals sent to the chronic pen Count and % of mortalities due to BRD Pen report: pen identified as BRD outbreak (yes/no) Pen report: pen-specific mass treatment (injectable or in-feed) Pen report: post-mortem and other diagnostic tests
Feedlot BRD treatment and mortality summary (analysis stratified for different classes of fed cattle)	Data Types 3, 6, 7, 8	Feedlot-level summary across all pen records for: Count and % of animals treated once for BRD Count and % of animals treated twice for BRD Count and % of animals treated three times for BRD Count and % of animals sent to the chronic pen Count and % of mortalities due to BRD Feedlot report: post-mortem and other diagnostic tests Feedlot summary: count and % of pens identified as BRD outbreaks Feedlot summary: count and % of pens that have received mass treatments (injectable or in-feed)
Regional BRD surveillance and research programs, policy changes and market drivers	Data Types 8 + 9	AMR surveillance: regional level temporal trends in BRD pathogen prevalence and antimicrobial susceptibility AMU surveillance: regional level temporal trends in AMU in feedlot cattle Published and/or practice-level research data Changes in government and/or industry policies/regulations influencing AMU Changes in market-driven policies influencing AMU

^1^ Types of data from Lane 1- Process Lane (outlined in Table 1).

The real-time BRD information can be in either a processed state, for example, data that have been collected, recorded, and analyzed, or can be in a ‘to be processed’ state, such as individual animal attributes and BRD treatment data flowing horizontally into both pen and feedlot treatment summaries. The information in the middle lane is continuously updated, but is also order-dependent, meaning the real-time BRD incidence and treatment response will impact what this information looks like at a given time. For example, the information could pertain to one individual calf, one purchase lot of calves, one pen, or the feedlot as a whole. The information will be different for a new individual calf, purchase lot, pen, and the feedlot over time. These summarized data flow up to the Information Processing Lane.

Data or information processes within the Information Lane (Table 2, Figure 1, Supplementary Figure S2) include the current BRD treatment plan (current drug stocks and records), individual animal attribute and management records, individual calf BRD treatment records (for real-time BRD incidence, treatment, and recovery/recurrence), pen-level BRD treatment summaries (across individual calf records within the pen), and the feedlot BRD treatment summary (across all pens in the feedlot). The additional external data that flow into the system are the retrospective, regional data from BRD surveillance and research programs, and any industry or government policies or regulations that impact AMU.

#### 2.2.3. Information Processing Lane (Top)

The Information Processing Lane includes the information assessment and subsequent decision points that the feedlot veterinarian will consider based on the processing of real-time information from the Information Lane (Figure 1, Supplementary Figures S1 and S2). The components of each information processing step for the BRD treatment plan are detailed in Table 3. Each of these information assessment steps (noted as stop signs in the IQ-VSMs) are based on internal data from the feedlot, which are housed in the feedlot management software that is linked to the clinic or external data (i.e., regional BRD surveillance and research data or policy data). 

There are two data sources for the pen- and feedlot-level information assessment steps (Table 3, Figure 1, Supplementary Figure S2). The first comes from internal feedlot data: (i) What was the risk of BRD? (ii) What was the pen-level success rate of the first and subsequent BRD treatment(s)? (iii) Was this a/were these pen-level outbreak(s)? (iv) What was the severity/scale of the BRD loss at the feedlot level? (v) Do the cumulative feedlot data align with the BRD treatment decisions? The second comes from external data: (i) Are there surveillance or research data that would change BRD treatment plans? (ii) Are there policy/regulatory changes or customer/market pressures that would change BRD treatment plans? 

Each of the internal and external information assessment steps was assigned a level of uncertainty (Table 3, legend in Supplementary Figure S2). Collectively, the processing of the internal and external information assessment steps leads to the current treatment plan value proposition (noted in the tombstone shape in the IQ-VSMs): does the assessment of the success of the current BRD treatment plan, relative to Process and Information Lane data, trigger a change to the BRD treatment plan? 

**Table 3:** Information Processing Lane: Processes and data flows from the Current State IQ-VSM (Figure 1, Supplementary Figure S2) that influence the control decision for potentially modifying a BRD treatment plan that might be used in a western Canadian commercial beef cattle feedlot production system.

Information Assessment	Type of Data	Explanation: Information Processing Components to Inform Decisions	Degree of Uncertainty (Low/Medium/High)
What was the risk of BRD?	Internal data	What is the relative proportion of high-risk calves entering the feedlot? Was the BRD risk assessment accurate (based on outcomes)? Was the response to metaphylaxis as expected?	Low
What was the pen-level success rate for 1st and and subsequent BRD treatment(s)?	Internal data	Did the individual treatment failure rate for first and subsequent treatment(s) exceed a user-defined threshold?	Moderate ^1^
Was this a pen-level outbreak?	Internal data	Does the pen-level incidence exceed a user-defined threshold to trigger a pen-level mass treatment intervention (all calves treated with injectable or in feed antimicrobials)?	Low
What was the severity/scale of the BRD loss at the feedlot level?	Internal data assessment	Does the feedlot-level incidence of BRD loss exceed the user-defined threshold for the assigned BRD risk category of the calves?	Low
Do the cumulative feedlot data align with the current BRD treatment decisions?	Internal data assessment	Do post-mortems, treatment records, or records of clinical signs suggest unexpected bacterial or viral disease? Are cases of BRD occurring when expected based on DOF and assigned BRD risk category?	Moderate ^2^
Are there surveillance or research data that would change BRD treatment plans?	External data assessment	Do regional surveillance data or new research suggest the need for, or an advantage to, an alternative treatment plan?	High
Are there policy/regulatory changes or customer/market pressures that would change BRD treatment plans?	External data assessment	Are there external pressures from domestic or international regulators that are advocating for changes in AMU? Are there external market pressures from retailers that are advocating for changes in AMU?	High
Current treatment plan: value proposition	Information processing that follows assessments	Does the assessment of the success of the current plan based on the processing of internal and external data trigger a change to the current BRD treatment plan?	N/A
Control decision: +/− change current feedlot BRD treatment plan and feedback loop	Decision	Update current BRD treatment plan based on feedlot level and external information	N/A

^1^ Possible contributors to uncertainty: BRD not detected fast enough, BRD treatment drug not appropriate for etiology, AMR, viral disease, calves are immunocompromised. ^2^ Possible contributors to uncertainty: most laboratory testing limited to BRD mortalities, limited laboratory testing in support of post-mortem examinations, limited ante mortem laboratory testing.

In the case of treating sick calves, the control decision represents a potential change to the pen- and feedlot-specific BRD treatment plan (i.e., the collation of BRD treatment protocols, designed by the feedlot veterinarian for pens within each feedlot—noted as the diamond shape in the IQ-VSMs). This decision point is represented by the dotted green feedback loop from the Information Processing Lane back down to the Information and Process Lanes. The cognitive task of decision-making is carried out by the veterinarian. The information processing could, therefore, include data on which drug(s) was/were used for initial and subsequent treatments and the pen-level responses of the sick, treated calves. In the cases of treatment failures, the veterinarian uses data to form an alternative hypothesis as to why treatment was not successful, with the potential for a subsequent change to the BRD treatment plan. The information on individual calves, pens, the feedlot, and external sources is processed and factored into the current BRD treatment plan that then feeds back down to the bottom two lanes with the potential for revisions to the plan.

### 2.3. Future State IQ-VSM

**Figure 2:**
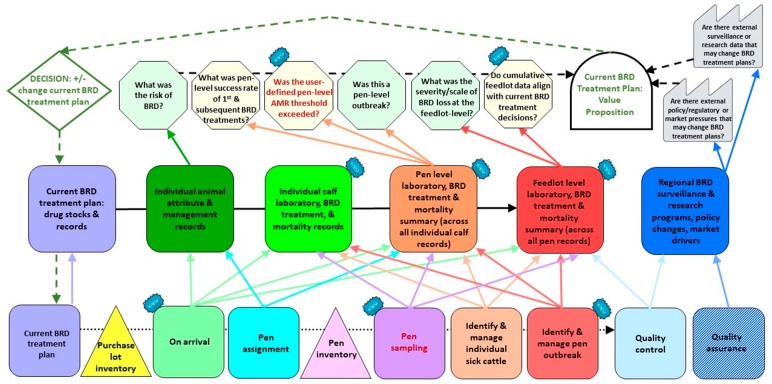
The simplified Future State IQ-VSM and associated opportunities for the integration of laboratory testing data, Kaizen—shown as blue cloudbursts (Table 4, Supplementary Tables S1–S3) for BRD treatment plans used in a western Canadian commercial beef cattle feedlot production system. The detailed Future State IQ-VSM is provided in Supplementary Figure S3. The bottom row is the Process Lane, the middle row is the Information Lane, and the top row is the Information Processing Lane. Dotted horizontal arrow: associated information (static, order-independent data). Solid arrows: processed/to be processed information (dynamic, order-dependent data updated in real-time). Dashed black arrow: information processing related to the control decision. Dashed green arrow: control decision feedback loop. ‘Pen sampling’ in red represents a new process.

In the bottom Process Lane of the Future State IQ-VSM (Table 4, Supplementary Table S1, Figure 2, Supplementary Figure S3), we identified three production processes that provide opportunities for respiratory sample collection for laboratory analysis for BRD pathogens and AMR (Kaizen, marked as blue cloudbursts): (i) On-arrival process, (ii) a new, added process of pen sampling, and (iii) the process to Identify and Manage a Pen Outbreak. Two of these production processes (on-arrival and identification and management of pen-level outbreaks) allow for the collection of samples at a time when the cattle are already moving through the processing facility. On-arrival sampling during initial processing and pen sampling at approximately 10–14 DOF entails collecting samples from a representative group of calves in a pen, regardless of their clinical BRD status. The pen sampling at 10–14 DOF would require selecting a subset of calves from a given pen and collecting samples from them in the feedlot handling facility outside of typical animal handling. 

Bacterial culture and antimicrobial susceptibility testing (AST) or metagenomic sequencing results from samples collected on arrival provide information about BRD pathogen and AMR phenotype or ARG detection from high-risk calves in purchase lots before antimicrobial metaphylaxis, sorting, and pen assignment [24,25,26,27]. At this time point, there have been fewer opportunities for contagious BRD pathogens to spread and infect purchase lot mates.

**Table 4:** Kaizen (opportunities for continuous improvement) identified for the Process Lane (production processes), Information Lane (BRD information processes), and Information Processing Lane (BRD information assessment and processing) of the Future State IQ-VSM (Figure 2, Supplementary Figure S3, Supplementary Tables S1–S3) for BRD treatment plans that might be used in a western Canadian commercial beef cattle feedlot production system.

IQ-VSM Lane	Kaizen
Process Lane	Kaizen 1: On-arrival process (representative sample of calves from a pen at arrival) Kaizen 2: Pen sampling (representative sample of calves from a pen at 10–14 days on feed) * Kaizen 3: Identify and manage pen outbreak
Information Lane	Addition of laboratory data from sampling to: Kaizen 1: Individual calf laboratory, BRD treatment, and mortality records Kaizen 2: Pen-level summary of individual laboratory, BRD treatment, and mortality records Kaizen 3: Feedlot-level summary of pen-level laborato-ry, BRD treatment, and mortality records
Information Processing Lane	Kaizen 1: Based on pen-level laboratory results, was the user-defined pen-level AMR threshold exceeded? Kaizen 2: Do cumulative feedlot-level data align with current BRD treatment decisions?

* New process introduced in Future State IQ-VSM.

In comparison, laboratory results from samples collected during the process of pen sampling (Table 4, Supplementary Table S1, Figure 2, Supplementary Figure S3) provide information about BRD pathogen and phenotypic AMR/ARG detection from high-risk calves 10–14 days after antimicrobial metaphylaxis, sorting, and pen assignment. By this time, contagious BRD pathogens and related AMR have had opportunities to spread and infect pen mates [24,27,28,29]. Importantly, sampling at this time can also detect AMR/ARGs that have developed as a result of selection pressure from antimicrobial metaphylaxis administered on arrival. Laboratory results from this second sampling time point could also be received early enough to precede the anticipated peak of first treatment for BRD within the sampled pen. 

Laboratory results from samples collected during the process of identification and management of pen outbreaks provide information about BRD pathogen and phenotypic AMR/ARG detection from either high-risk or low-risk feedlot cattle during a presumptive pen outbreak of BRD. This third sampling time point occurs days to weeks after antimicrobial metaphylaxis, sorting, and pen assignment. 

In the middle Information Lane of the Future State IQ-VSM (Table 4, Supplementary Table S2, Figure 2, Supplementary Figure S3), we identified three information processes as Kaizen. These provide opportunities to integrate and compile real-time laboratory data at multiple levels: (i) Individual Calf BRD Treatment Records, (ii) Pen-level BRD Treatment Summary (across all individual calf records within each pen), and (iii) Feedlot-level BRD Treatment Summary (across all pen records within the feedlot). 

In the Information Lane, summarized pen-level laboratory data are compared against the user-defined pen-level AMR thresholds that the veterinarian previously entered into the feedlot management software (bottom Process Lane). An example AMR threshold for the pen sampling time point could be exceeded when (i) *Mannheimia haemolytica* is found in 10/20 respiratory samples from a pen of high-risk calves, and (ii) these also carry phenotypic AMR or ARGs against the first-choice BRD treatment antimicrobial in the current BRD treatment protocol. If the user-defined pen-level AMR threshold is exceeded for any sampling points (on arrival, pen, or outbreak), the feedlot management software would send an alert notification to the veterinarian. The veterinary practice could customize these thresholds within their feedlot management software. 

In the top Information Processing Lane of the IQ-VSM, two information processing steps were identified for Kaizen (Table 4, Supplementary Table S3, Figure 2, Supplementary Figure S3) as they provided an opportunity to analyze and then utilize both pen-level and feedlot-level laboratory data in the veterinarian’s decision-making process: (i) Based on pen-level laboratory results, was the user-defined pen-level AMR threshold exceeded (yes or no)?, and (ii) Do cumulative feedlot-level data align with current BRD treatment decisions? Importantly, in the proposed Future State IQ-VSM, laboratory results are not used to inform individual calf-level BRD treatment decisions; rather, they are used to provide pen-specific and feedlot-specific BRD pathogen and AMR results that subsequently inform pen-specific and feedlot-specific decisions about BRD treatment plans.

The evaluation of each Kaizen using the three information quality dimensions—granularity, frequency, and accuracy [11]—required the definition of how each dimension applied to the three IQ-VSM lanes in the context of laboratory testing for BRD management in western Canadian commercial feedlots:

1. Granularity: The degree of resolution for which the considered information is available. The granularity influences the efficacy of control methods, as it defines the representation of the real-life phenomenon [11]:
  - Process Lane: The number of calves in a purchase lot or pen that can be sampled at any of the three sampling time points as well as the number of purchase lots or pens sampled per feedlot in the current fall run of calves entering the feedlot;
  - Information Lane: The 95% confidence intervals (CIs) for the resulting proportions of reported laboratory outcomes for individual pens (ARGs or phenotypic AMR for specific BRD pathogens) and how these data vary across pens within the feedlot;
  - Information Processing Lane: The number of pens sampled and precision of the resulting 95% CIs that are sufficient to inform subsequent decisions on BRD treatment protocols at the pen and feedlot level relative to other information sources.
2. Frequency: The time interval in which the information is acquired or has been updated [11]. Delayed information could reduce or eliminate the value of data for time-sensitive decisions:
  - Process Lane: (i) timing of the sample collection (relative to when calves arrive), (ii) number of times a pen is sampled (individual pens may be sampled more than once), and (iii) not all pens will be sampled. The veterinarian will determine the pen sampling rate in order to select which pens will be sampled and what proportion of pens will be sampled throughout the current run;
  - Information Lane: Timing of receipt and upload of laboratory results into the feedlot data management system;
  - Information Processing Lane: Laboratory results are available in sufficient time for analysis to inform decisions on the appropriateness of current BRD treatment plans for (i) the sampled pen(s), (ii) other similar but non-sampled pens during the current run, (iii) and/or decisions regarding the appropriateness of feedlot-level treatment plans for the current and subsequent years.
3. Accuracy: The degree to which the obtained information represents the real-life phenomenon. [11]. Discrepancies are related to the measurement unit described by the IQ dimension granularity and include all possible influencing factors responsible for deviations:
  - Process Lane: (i) the degree to which the sample received by the laboratory can be analyzed to reflect the presence of BRD pathogens and the AMR status (e.g., the quality of the collected sample and shipping efficiency/impacts of shipping delays), and (ii) limitations of the laboratory procedures for bacterial isolation and AST or metagenomic sequencing and bioinformatics;
  - Information Lane: (i) completeness of the list of BRD pathogens and types of ARGs or phenotypic AMR identified and reported by the laboratory, and (ii) what is known regarding the sample-level diagnostic sensitivity and specificity of these tests;
  - Information Processing Lane: (i) How does the resulting information reflect the true antimicrobial susceptibility of BRD pathogens in fall-placed high-risk calves at the pen and feedlot level for the current production run? (ii) How can this information be used to inform decisions on the appropriateness of current BRD treatment protocols?

The specific, extensive evaluation of each of the identified Kaizen from the three levels and the three information quality dimensions (Supplementary Tables S1–S3) provided a detailed understanding of how laboratory detection of BRD pathogens and both phenotypic AMR and metagenomic ARGs could fit into the assessment of antimicrobial treatment plans as part of the BRD management strategy in western Canadian feedlots. The information quality matrices (Supplementary Tables S1–S3) helped us understand the precision, quality, and timeliness of laboratory BRD pathogen and AMR information required to make informed decisions regarding the appropriateness of the current BRD treatment plan.

## 3. Discussion

This Current and Future State IQ-VSMs for AMU for BRD treatment in western Canadian commercial feedlots provided visual representation and explicit documentation of the data inputs and processing steps within this complex livestock production system. This represents a novel application of information quality value stream mapping to such a system, specifically to understand how laboratory testing can be implemented to support pen- and feedlot-specific antimicrobial decisions for BRD treatment plans. This visual representation, supported by detailed documentation, provides a robust plan for the integration of cohort-level laboratory testing to support AMS in feedlot production that could include traditional phenotypic AST or metagenomic sequencing to detect BRD pathogens and ARGs. The IQ-VSM also illustrates the potential for the resulting laboratory data to be incorporated into national feedlot surveillance programs for AMR.

In the context of BRD management in commercial feedlots, mapping the production system in this way is unique given that animals in a feedlot are not manufacturing entities. In this case, thousands of animals of varying sizes, ages, weights, and risk for BRD move through the production system with a variable and extended time from entry to exit from the feedlot. Animal health, and specifically BRD information flows, are highly complex. Databases and BRD treatment and management algorithms used in feedlot operations are proprietary [14,30,31,32,33] and increasingly specialized given the size of present-day feedlots [26], the highly competitive nature of the business, narrow profit margins, and the impact of IR4.0 digital technologies on livestock production [34]. The knowledge to construct the Current State IQ-VSM and then conduct the value stream analysis to identify the continuous improvement Kaizen leveraged the feedlot expertise and experience of the project team [4,24,25,27,35], which includes skilled research veterinarians that frequently interact with feedlot veterinarians and veterinary technical staff, feedlot staff, and managers. The researchers have also worked with the core feedlot veterinary practices and veterinarians in western Canada. Together, these partnerships continue to contribute to national AMR and AMU surveillance for the feedlot [14,36,37] and cow-calf sectors [38,39,40,41]. 

Ultimately, this process allowed us to document how BRD information flows through the feedlot system and translates into decisions about BRD treatment plans. We have mapped how to integrate not only sampling but also the subsequent laboratory data from individual animals into this system designed to inform pen- and feedlot-level assessment of BRD rates. This will allow us to better understand how and where to bring in new testing strategies such as pen-level sampling [24,27], phenotypic AST [27], and metagenomic sequencing for the detection of BRD pathogens and ARGs [25,42]. 

A fundamental element of value stream mapping is the focus on continuous quality improvement to production practices to reduce loss [5]. In this light, using the IQ-VSM for BRD management that integrates laboratory testing to support AMS is comparable to the quality improvement approach for the implementation of stewardship programs in human medical settings. McGregor et al. [43] discussed how AMS relies on a set of strategies to improve the clinical prescribing of antimicrobials by physicians and subsequent use by patients to improve patient safety, which shares common elements with quality improvement. Stewardship programs in human hospitals tend to focus on the implementation of strategies into routine clinical care, such as education, guidelines, and team-based review of prescribing. There is a broader body of literature on the use and effectiveness of continuous quality improvement on professional practice and health care outcomes [44,45].

An important distinction is that continuous quality improvement for AMS in the human health care system focuses on outcomes at the level of individual patients. Conversely, livestock production is commonly focused on animal health and welfare outcomes at the group level. This is particularly evident in the case of BRD, AMR, and the integration of laboratory testing to support AMS in the case of the IQ-VSM for feedlot cattle. Current laboratory testing technologies fit best with a pen-level assessment of BRD and AMR results given the expected turnaround time of 3 to 7 days [4] and the need to make changes to BRD treatment plans at the level of the pen or feedlot. In many cases, the laboratory information will be expected to precede the peak of BRD cases and inform treatment of these subsequent cases from the same pen; in pens where it does not, the information can be used to inform treatment plans for cattle with a similar risk profile.

Our first objective centered on the application of the IQ-VSM to BRD management as a continuous improvement tool. The BRD treatment plan is the collective suite of pen- and feedlot-specific treatment protocols for different situations that are designed by a feedlot veterinarian and provided to the feedlot so they can treat cattle that get sick with BRD with the goal of managing animal health and welfare as well as meet expectations for AMS. The information quality aspects of the Current State IQ-VSM build on the published work of IQ-VSMs in an information management context [9,10,11]. In the context of a western Canadian feedlot, the IQ-VSM incorporates the concept of information flows, data management, and storage of retrospective data in proprietary and specialized feedlot management software to inform BRD treatment protocols. In short, the Current State VSM (i) provides a visualization of the steps involved in BRD treatment and management in commercial feedlots in western Canada and (ii) illustrates potential opportunities to improve the production system. 

Our second objective used Kaizen identification within the Future State IQ-VSM to create a common understanding of how information from laboratory testing as well as BRD morbidity, mortality, and feedlot wastage/chronicity rates can inform the decision-making process for pen- and feedlot-specific BRD treatment protocols based on current state feedlot production processes. The Future State IQ-VSM provides (i) a visualization of the steps involved in BRD treatment and management in commercial feedlots typical of western Canada, (ii) an illustration of opportunities that could improve evidence supporting pen- and feedlot-specific BRD treatment protocols, and (iii) a demonstration of how we can improve and expand veterinary laboratory testing support for evidence-informed AMU in large commercial feedlots typical of western Canada.

We were able to articulate where laboratory data could be integrated for continuous improvement of BRD management within feedlot production. This allowed us to understand, detail, and evaluate how this integration of laboratory data for BRD pathogen and AMR detection could support AMS. By incorporating laboratory data, whether it be culture and phenotypic AST or metagenomic pathogen and ARG detection, feedlot veterinarians would have tools to understand BRD and AMR transmission at the pen and feedlot levels. The assessment of these data would then allow them to determine the appropriateness of the current pen- and feedlot-specific BRD treatment plans. This is important because it highlights the ability for pen-specific data processing and decision-making that allow for customized BRD treatment plans for individual pens within individual feedlots.

This IQ-VSM provides a robust framework to understand not only where sample collection and testing can be used within production processes but also how the data can be collated and assessed to specifically inform BRD treatment plans that are a pillar of antimicrobial strategies for the management of this important disease complex for feedlot cattle. The Future State IQ-VSM provides a visual representation with detailed documentation of what the feedlot veterinarian may be required to implement if regulatory policies emerge that require the justification of the use of certain antimicrobials with (i) *ante mortem* (live animal) sampling and laboratory testing evidence of BRD pathogens, and (ii) the presence/absence of phenotypic AMR or ARGs.

International policy is moving towards the requirement for laboratory testing to support the prophylactic use of antimicrobials in food animal production [2,46]. McDonald’s, one of the largest customers for Canadian beef production, is moving towards a similar requirement for the use of antimicrobials important to human health [47]. As a result, there may come a time in the near future when integration of laboratory testing into BRD management in western Canadian feedlot production becomes a regulatory and/or market requirement. For example, as of 25 February 2019, a new regulation in the Canadian province of Québec [48] requires laboratory evidence in order to use a category I antimicrobial, designated as “very high importance” by Health Canada [49], in a food-producing animal for “curative purposes”. Laboratory tests (e.g., culture and AST) are required to show that the infection is not treatable by a lower category antimicrobial [48]. The regulation further prohibits the use of category I antimicrobials for disease prophylaxis in food animals. The current McDonald’s policy specifically points to the use of macrolides in food animals [47]. Macrolides (Canadian Category II—High Importance [49], WHO Critically Important Antimicrobial [50]) and tetracyclines (Canadian Category III—Medium Importance [49], WHO Highly Important Antimicrobials [50]) are common antimicrobials used for BRD management (metaphylaxis and treatment) in western Canadian feedlots [14] with efficacy [51]. This emphasizes the need for the use of antimicrobials in BRD management to strive for optimal AMS. The use of IQ-VSMs as a continuous improvement tool provided a detailed understanding of BRD management to identify where laboratory testing could be implemented to support AMS.

This IQ-VSM did not consider the validation of pen- and feedlot-specific BRD treatment plans via subsequent laboratory testing of BRD treatment failures, chronic BRD cases, or post-mortems of BRD mortalities. Most of these animals would have been treated multiple times with antimicrobials. Laboratory test results would not be informative for assessing the appropriateness of the first or likely even second treatment drug within the BRD treatment plan. The antimicrobials recommended for first BRD treatments will be specific for treating acute BRD in protocols provided by the consulting veterinarians and will be different from antimicrobials recommended for subsequent repeat treatments.

Our proposed testing strategy is based on the collection and testing of guarded deep nasopharyngeal swabs (DNPS) from feedlot calves. Most western Canadian feedlots have animal handling systems with chutes that contain neck extenders to facilitate DNPS sample collection. Highly skilled and experienced veterinary technologists and feedlot staff can efficiently collect DNPS within this system for submission to diagnostic laboratories for testing with minimal disruption to animal flow in the handling system. Published studies reporting the agreement between guarded DNPs and tests on deeper samples, such as bronchoalveolar lavage (BAL), focus on samples taken from calves with clinical BRD and describe inconsistent results. Some papers demonstrated substantial to almost perfect agreement [52,53,54] with another reporting moderate agreement [55]. Such studies examined agreement between DNPS and BAL results for the actual pathogens present and causing disease in the individual sick animals that were sampled.

Interestingly, this older study found that when DNPS and BAL results were compared at the group level, the isolation rates of the BRD pathogens were quite similar and the authors concluded that DNPS samples could be reliably used for research purposes to give a useful estimate of the pulmonary microbial flora in groups of feedlot calves with BRD [55]. The goal of this proposed testing strategy is to provide pen- and feedlot-level BRD pathogen and AMR data by collecting and testing DNP samples from a representative group of calves from pens at arrival and two weeks into the feeding period, regardless of clinical BRD status. These data are then processed along with BRD incidence and treatment data, as well as overall mortality records, to inform the decisions at the top of the IQ-VSM about pen- and feedlot-specific BRD treatment protocols.

The use of DNPS from healthy animals is both efficient for collection and effective at providing laboratory evidence for the bacterial BRD pathogens and their associated AMR that are circulating in all animals at these two time points, and the ability to assess how these change after the administration of antimicrobial metaphylaxis and entry into the post-metaphylaxis interval. Agreement with BAL is not a consideration for animals that do not have clinical BRD, as we would not expect them to have bacterial pathogens in their lungs. As such, the agreement between the results from DNPS and specific pathogens causing disease in a given sick animal is not required because the intent is not to understand test results at the level of each individual animal or specifically in animals with clinical BRD. Further, the collection of *ante mortem*, endoscopy-guided BALs or transtracheal washes [56] from cattle in a commercial western Canadian feedlot is not feasible or practical for routine BRD testing.

We acknowledge that the implementation of such a laboratory testing strategy would come at a cost to the feedlot operator and feedlot veterinarians and veterinary staff. Substantial coordination would be required to facilitate the collection of samples from a subset of calves as they enter the feedlot and again approximately two weeks into the feeding period. These costs would be financial (e.g., shipping and laboratory testing of samples), human resources (e.g., veterinary and/or feedlot staff for sample collection, packaging, and shipment), and time to pull animals from pens and put them through the animal handling facility for sample collection.

Ultimately, these items would need to be addressed through consultation with the veterinarians, veterinary technical staff, and feedlot owners/managers/staff. Any new strategy would need to show demonstrable benefits for feedlots in terms of animal health and welfare and overall production economics. It may well be that in the future, laboratory testing to justify AMU for BRD management could be required with changes to international regulations [46] or industry requirements [47]. Consultation with feedlot veterinarians and owners/managers represents an important area for future research.

Such testing strategies are feasible and effective in the real-world setting of a western Canadian feedlot. We have demonstrated initial success with both phenotypic [24,27] and metagenomic testing of respiratory samples [25] in field settings. At present, CIPARS works with feedlot veterinary clinics to collect respiratory samples from commercial feedlots in western Canada and test for phenotypic AMR in BRD pathogens, both at arrival and later in the feeding period [37]. Other applied research in commercial western Canadian feedlots in collaboration with feedlot veterinarians and CIPARS will further examine the utility of this laboratory testing strategy.

The IQ-VSM focused on using pen- and feedlot-level data to inform pen-specific treatment plans rather than treatment decisions for individual animals or metaphylaxis protocols. We approached this value stream mapping project with the understanding that existing laboratory testing is limited to tests that take, at a minimum, days to provide results after sample collection [4,12]. This is in part due to the sheer distance between the feedlot, veterinary clinic, and diagnostic laboratory. Even if tests could be completed within minutes to hours, they are only available at the laboratory, not chute-side or in the veterinary clinic. It takes a minimum of 1–2 days to get samples from the feedlot to the diagnostic laboratory. At this time, no sufficiently sensitive, commercially available options for field testing exist that produce results within minutes to inform individual animal drug selection for treatment or metaphylaxis. As a result, the proposed testing strategy is not intended to inform or assess the effectiveness of individual animal testing on individual BRD treatment outcomes.

The Future State IQ-VSM demonstrates how data from the testing of a sample of individual animals can be used to assess the appropriateness of pen- and feedlot-specific treatment plans. The lack of current population-level studies assessing the links between AMR in bacterial BRD pathogens and clinical outcomes after antimicrobial treatment represents an important area for future research. There are only a few studies that assessed the link between AMR and clinical BRD outcomes at the level of the individual calf, with little evidence to support a link [57,58,59,60,61]. Other large studies did not assess this specific outcome [26,62,63,64,65].

Our recent study on a large trial of feedlot cattle identified examples of both individual calf bacterial BRD culture and phenotypic AMR results, as well as the pen prevalence of BRD pathogen and AMR taken on arrival and at two weeks on feed, were linked to the risk of clinical BRD and AMR in bacterial isolates at the time of BRD diagnosis [27]. There was an increased risk of macrolide- or tetracycline-resistant BRD bacteria at treatment, which is associated with an increasing pen-level prevalence of macrolide- or tetracycline-resistant bacteria from samples at arrival and two weeks on feed. The evidence suggests that our proposed testing strategy could be useful for understanding population-level clinical BRD outcomes. Applied research using metagenomic testing of respiratory samples is ongoing [25,42].

## 4. Methods

The production system for this case study was a typical western Canadian commercial beef feedlot, and the product was the BRD treatment plan [5]. The BRD treatment plan includes the collective suite of BRD treatment protocols for different situations that are designed by a feedlot veterinarian and provided to the feedlot staff to treat calves that get sick with BRD. The goal of the BRD treatment plan is to minimize impacts on animal health and welfare, reduce economic loss from BRD morbidity/mortality, and meet the consumer and regulatory expectations for practicing AMS. In the case of the IQ-VSM, the ‘customer’ was the feedlot veterinarian, who is the one responsible for making decisions about the BRD treatment plan [5]. The value stream mapping activity was inclusive of the processes and the people involved in managing BRD and treating sick calves as well as the information and data associated with this production system.

The IQ-VSM [9,10,11] begins at the time a purchase lot of beef calves arrives at the feedlot and ends with a healthy calf ready for sale or a dead calf that did not respond to BRD treatment protocols. The production processes included in-house BRD treatment protocols, on-arrival processing for BRD management, pen assignment, identification of individual sick calves by the people involved in handling the animals (e.g., pen riders), pen outbreak identification and response, and quality control and quality assurance processes (Figure 1 and Figure 2 and Supplementary Figures S2 and S3).

The information quality aspects of the IQ-VSM (Figure 1) incorporated the concept of information flows, data management, and storage of the retrospective data to inform BRD treatment plans. These came from IR4.0 aspects of digital technologies that inform production systems [66,67] with information flow and quality aspects [9,10,11]. The bottom Process Lane (Figure 1 and Figure 2 and Supplementary Figures S2 and S3) included the information resources and static data for production processes in a series of process boxes and inventory triangles that did not change in real time. The middle Information Lane (Figure 1 and Figure 2 and Supplementary Figures S2 and S3) represented sinks that collected and organized information from each production process in the bottom lane and transmitted it for processing in the top lane. These sinks, represented by rectangles, were real-time, order-dependent information flows that are continuously updated, represented by rectangles. The top Information Processing Lane (Figure 1 and Figure 2 and Supplementary Figures S2 and S3) represented information assessment steps (stop signs), collective information processing (tombstone), and a final control decision (diamond), with subsequent feedback into the middle and bottom lanes of the system based on the processing of dynamic, real-time information flowing from the middle lane. Each information assessment step was assigned a level of uncertainty based on researcher knowledge of the Canadian feedlot industry and the current Canadian state of AMR/AMU surveillance, AMU and stewardship policies, and the consumer marketplace (Table 3, Figure 1 and Figure 2 and Supplementary Figures S2 and S3).

We created a Current State IQ-VSM (Figure 1 and Supplementary Figure S2) through iterative discussion with feedlot researchers on our team to visualize the steps involved in BRD treatment and management in typical commercial western Canadian feedlots. The Current State IQ-VSM scope included the current BRD treatment plans typical for a western Canadian feedlot. We then developed the Future State IQ-VSM (Figure 2 and Supplementary Figure S3) that incorporated the identified Kaizen [5]. These Kaizen represent opportunities to make continuous improvement changes to pen- and feedlot-specific BRD treatment plans by incorporating bacterial culture and AST and/or metagenomic sequencing for the detection of BRD pathogens and associated phenotypic AMR or ARGs into the BRD management system of a feedlot.

Once identified, each production process, information process, and information processing step identified as Kaizen was further examined with the lens of information quality dimensions: the granularity, frequency, and accuracy of information (Table 4, Supplementary Tables S2 and S3) [11]. This allowed us to map how the integration of laboratory testing of respiratory samples at western Canadian commercial feedlots could continuously improve BRD treatment plans within feedlots and support evidence-informed AMU. Granularity is the degree of resolution at which the considered information is available. Frequency describes the time interval in which the information is acquired or has been updated. Delayed information could reduce or eliminate the value of data for time-sensitive decisions. Accuracy describes the degree to which the obtained information represents a real-life phenomenon.

Purchase lots of beef cattle entering western Canadian feedlots are defined by risk categories for BRD based on their (i) relative age and weight, (ii) source, and (iii) time of year of entry. For the purposes of these VSMs and the information quality matrix, we described high- and low-risk categories for BRD. These two risk categories also generally represent the two largest groups of cattle entering western Canadian feedlots. High-risk calves typically include recently weaned, auction market sourced, fall-placed calves, while low-risk calves are typically older yearling beef cattle.

## 5. Conclusions

The implementation of the IQ-VSM to understand BRD management in western Canadian commercial feedlot production is unique as this complex biological system has numerous, heterogeneous information inflows coming from thousands of beef cattle of varying age, weight, and risk for BRD, with variable and extended entry-to-exit times. The IQ-VSM provided a valuable tool to visualize and document how BRD pathogen and AMR information fit into the risk-based management system for BRD in western Canadian feedlots. The identified opportunities for continuous improvement showed where laboratory testing and information can be integrated into the system, how the information quality is considered, and ultimately, how laboratory data can be used to support AMS by assessing the appropriateness of current BRD treatment plans and informing future pen-specific BRD treatment plans.

## Data Availability

The original contributions presented in the study are included in the article/Supplementary Material, further inquiries can be directed to the corresponding author.

## Supplementary Materials

The following supporting information can be downloaded at https://www.mdpi.com/article/10.3390/antibiotics13090903/s1, Table S1: The information quality matrix for the Kaizen (opportunities for continuous improvement) identified for the Process Lane of the future state Information Quality Value Stream Map (IQ-VSM) (Figure 2, Supplementary Figure S3) of Bovine Respiratory Disease (BRD) treatment plans used in a western Canadian commercial beef cattle feedlot production system; Table S2: The information quality matrix for the Kaizen (opportunities for continuous improvement) identified for the Information Lane of the future state IQ-VSM (Figure 2, Supplementary Figure S3) of BRD treatment plans used in a western Canadian commercial beef cattle feedlot production system.; Table S3: The information quality matrix for the Kaizen (opportunities for continuous improvement) identified for the Information Processing Lane of the future state IQ-VSM (Figure 2, Supplementary Figure S3) of BRD treatment plans used in a western Canadian commercial beef cattle feedlot production system; Figure S1: Generic IQ-VSM; Figure S2: Detailed Current State IQ-VSM for BRD Treatment Plan; Figure S3: Detailed Future State IQ-VSM for BRD Treatment Plan. References [24,27] are cited in Supplementary Materials.
