# Supplementary material for: Opportunities for Laboratory Testing to Inform Antimicrobial Use for Bovine Respiratory Disease: Application of Information Quality Value Stream Maps in Commercial Feedlots
Source: Antibiotics (Basel). 2024 Sep 21;13(9):903. doi: 10.3390/antibiotics13090903 (PMC11428555; doi:10.3390/antibiotics13090903)
Supplement: Supplementary file 1 [file antibiotics-13-00903-s001.zip › antibiotics-3118754-supplementary - english editing-corrections.20Sept2024.pdf]

Opportunities for Laboratory Testing to Inform Antimicrobial Use for Bovine Respiratory Disease: Application of Information Quality Value Stream Maps in Commercial Feedlots

Simon J. G. Otto [1,2,*], Colleen M. Pollock [1,3], Jo-Anne Relf-Eckstein [1,3], Lianne McLeod [3] and Cheryl L. Waldner [3,*]

[1] HEAT-AMR (Human-Environment-Animal Transdisciplinary Antimicrobial Resistance) Research Group, School of Public Health, University of Alberta, Edmonton, AB T6G 1C9, Canada, ; colleen.pollock@usask.ca (C.P.); jer128@mail.usask.ca (J.-A.R.-E.)
[2] Healthy Environments Thematic Area Lead, School of Public Health, University of Alberta, Edmonton, AB T6G 1C9, Canada
[3] Department of Large Animal Clinical Sciences, Western College of Veterinary Medicine, University of Saskatchewan, Saskatoon, SK S7N 5B4, Canada; lianne.mcleod@usask.ca
* Correspondence: simon.otto@ualberta.ca (S.J.G.O.); cheryl.waldner@usask.ca (C.L.W.)

Supplementary Tables

Supplementary tables include detailed descriptions of the Kaizen identified for each lane of the information quality value stream map (IQ-VSM) (Table 4) along with the information quality dimensions for each:

- **Granularity**: the degree of resolution for which the considered information is available
- **Frequency**: the time interval in which the information is acquired or has been updated
- **Accuracy**: the degree to which the obtained information represents the real-life phenomenon

The cell shading colours used on each of the tables are matched to the corresponding items on the IQ-VSM (Figure 2, Supplementary Figure S3).

Supplementary Table S1. The information quality matrix for the Kaizen (opportunities for continuous improvement) identified for the Process Lane of the future state Information Quality Value Stream Map (IQ-VSM) (Figure 2, Supplementary Figure S3) of Bovine Respiratory Disease (BRD) treatment plans used in a western Canadian commercial beef cattle feedlot production system.

**Process Lane Kaizen: production processes**	**Granularity**	**Frequency**	**Accuracy**
**KAIZEN 1**<br><br>**ON-ARRIVAL PROCESS**<br><br>The **'on-arrival' process** provides the **1st** continuous improvement **(Kaizen) opportunity** for **collection of deep nasal pharyngeal samples (DNPS)** for **laboratory testing** (metagenomic or culture/AST data).<br><br>DNPS could be collected from a **representative sample of calves** from a **sample of purchase lots designated as high risk (HR) for BRD, <u>prior to</u> antimicrobial metaphylaxis, sorting, & pen assignment.** At this sample time point, there would have been **fewer opportunities for**	The factor that most directly influences **granularity of sample collection** is **sample size**.<br><br>The veterinarian would determine **(i) how many calves within each HR purchase lot** would be sampled* and **(ii) how many HR purchase lots within the current run** are to be sampled*.<br><br>The veterinarian could also **consider how both the cost and time required** to collect the DNPS **affects the sample size decision**.<br><br>Note: laboratory test results will follow in the Information Lane.<br><br>**Research will inform veterinary decisions on the level of certainty*	Numerous purchase lots continuously arrive at the feedlot throughout the 'fall run'; however, only **a portion of HR purchase lots would be sampled at the time of 'on-arrival' processing** (sample time point 1).<br><br>The veterinarian would determine **the sampling rate** and other criteria needed to select **which HR purchase lots will be sampled.** For example, a veterinarian might sample every 5th or every 10th HR purchase lot as they arrive and are processed at the feedlot.	As sample size influences granularity, **changing sample size influences the accuracy of what is measured** in the samples collected 'on-arrival' (sample time point 1).<br><br>The veterinarian would determine an **appropriate sample size to reflect the anticipated purchase lot-level prevalence of AMR for specific BRD pathogens** present in the respiratory samples.<br><br>In addition, having the **DNPS sampling process carried out by trained veterinary professionals** (registered veterinary technologists or veterinarians) **following a standard operating procedure (SOP)** to collect, label, and ship the temperature-controlled samples within 24 hours of collection would **increase the accuracy of what is**

**Process Lane Kaizen: production processes**	**Granularity**	**Frequency**	**Accuracy**
**contagious BRD pathogens to spread & infect purchase lot mates**.	*expected with different sample sizes.*		**measured** in the 'on-arrival' samples.
**KAIZEN 2**<br><br>**PEN SAMPLING**<br><br>The '**pen sampling' process** provides a **2nd** continuous improvement **(Kaizen) opportunity** for **collection of DNPS** for **laboratory testing** (metagenomic or culture/AST data).<br><br>DNPS could be collected from a **representative sample of calves** from a **sample of pens designated as HR for BRD, ~ 10 to 14 days after antimicrobial metaphylaxis, sorting, & pen assignment.** By this time, there have been **many opportunities for contagious BRD pathogens to infect pen mates**. This	The factor that most directly influences **granularity of sample collection** is **sample size.**<br><br>The veterinarian would determine **(i) how many calves within each HR pen** are to be sampled* and **(ii) how many HR pens within the current run** are to be sampled*.<br><br>The veterinarian could also **consider how both the cost and time required** to collect the DNPS **affects the sample size decision.**<br><br>Note: laboratory test results will follow in the Information Lane.<br><br>**Research will inform veterinary decisions on the level of certainty*	Numerous HR pens are established throughout the 'fall run'; only a portion of HR pens would be sampled **shortly after arrival for 'pen sampling'** (sample time point 2).<br><br>The veterinarian would determine the **feasibility of sample collection at this sample time point**, based on availability of labor and processing facilities.<br><br>The veterinarian would then determine the **appropriate timing of sample collection relative to feedlot arrival, the post-metaphylactic interval, and anticipated peak of BRD cases**.<br><br>Lastly, the veterinarian would determine **the pen-level**	As sample size influences granularity, **changing sample size influences the accuracy of what is measured** in the samples collected at 'pen sampling' (sample time point 2).<br><br>The veterinarian would determine an **appropriate sample size and timing of sampling collection to reflect the anticipated pen-level prevalence of AMR for specific BRD pathogens** present in the respiratory samples.<br><br>In addition, having the **DNPS sampling process carried out by trained veterinary professionals** (registered veterinary technologists or veterinarians) **following a standard operating procedure (SOP)** to collect, label and ship the cooled samples within 24 hours of collection would **increase the**

**Process Lane Kaizen: production processes**	**Granularity**	**Frequency**	**Accuracy**
sampling should **occur before the anticipated peak of 1st treatment for BRD** for the sampled pen.	*expected with different sample sizes.*	**sampling rate** to select **which HR pens will be sampled.** For example, a veterinarian might select and sample every 5th or every 10th HR pen as they are established at the feedlot.	**accuracy of what is measured** in the samples collected at 'pen sampling'.

**Process Lane Kaizen: production processes**	**Granularity**	**Frequency**	**Accuracy**
**KAIZEN 3**<br><br>**IDENTIFY & MANAGE PEN OUTBREAK**<br><br>The **process to 'identify & manage a pen outbreak'** provides a **3rd** continuous improvement **(Kaizen) opportunity** for collection of **DNPS** for **laboratory testing** (metagenomic or culture/AST data) <u>if the outbreak pen is treated with injectable antimicrobials or re-vaccinated</u>.<br><br>**DNPS could be collected from a representative sample of cattle from outbreak pens** as they are processed through the hospital facility for **fever assessment, BRD treatment, or re-vaccination.**	The factor that most directly influences **granularity of sample collection** is **sample size**.<br><br>The veterinarian would determine **how many cattle within each outbreak pen** are to be sampled*.<br><br>The veterinarian could also **consider how both the cost and time required** to collect the DNPS **affects the sample size decision**.<br><br>Note: laboratory test results will follow in the Information Lane.<br><br>**Research will inform veterinary decisions on the level of certainty expected with different sample sizes.*	As this **third opportunity for DNPS sample collection occurs sporadically, the veterinarian could collect DNPS for laboratory testing from as many outbreak pens as feasible as cattle are processed through the hospital facility** for fever assessment, BRD treatment, or re-vaccination (sample time point 3).	As sample size influences granularity, **changing sample size influences the accuracy of what is measured** in the 'pen outbreak' samples, collected at sample time point 3.<br><br>The veterinarian would determine an **appropriate sample size to reflect the anticipated 'outbreak' pen-level prevalence of AMR for specific BRD pathogens** present in the respiratory samples.<br><br>In addition, having the **DNPS sampling process carried out by trained veterinary professionals** (registered veterinary technologists or veterinarians) **following a standard operating procedure (SOP)** to collect, label and ship the cooled samples within 24 hours of collection would **increase the accuracy** of what is measured in the 'pen outbreak' samples.

Supplementary Table S2. The information quality matrix for the Kaizen (opportunities for continuous improvement) identified for the Information Lane of the future state IQ-VSM (Figure 2, Supplementary Figure S3) of BRD treatment plans used in a western Canadian commercial beef cattle feedlot production system.

**Information Lane Kaizen: BRD information processes**	**Granularity**	**Frequency**	**Accuracy**
**KAIZEN 1**<br><br>**INDIVIDUAL CALF LABORATORY, BRD TREATMENT, AND MORTALITY RECORDS**<br><br>**Individual calf laboratory, BRD treatment, and mortality records** contribute to an information process that **was identified as Kaizen,** as **laboratory test results (metagenomic or culture/AST data) could be <u>uploaded</u> <u>at the individual calf level</u>**.<br><br>Note: in the proposed future state VSM, laboratory results would not be used to inform individual calf-level BRD treatment decisions.	**ARGs/mutations or phenotypic AMR for specific BRD pathogens** would be reported by the laboratory. **Individual calf laboratory test results** could be **uploaded** into the feedlot management software and **outcomes reported** for each of the available sampling time points: 1, 2 or 3.<br><br>**A potential strength of long-read metagenomics compared to culture/AST data is the degree of resolution.** Long-read metagenomics has the potential to assess (in a single sample) AMR in (i) more types of bacteria, (ii) more types of genes and mutations, from (iii) more classes of antimicrobials than AST.<br><br>**The granularity of culture/AST data is influenced by**: (i) the	As sampling protocols, shipping time, laboratory processing time, laboratory reporting time and information communications technologies improve, the time interval from sample collection to acquired laboratory results will decrease.<br><br>**Uploading of laboratory results for each sampled calf** into the feedlot management software **could occur as the laboratory results become available**.<br><br>The **laboratory test results in each individual calf record would be reported with the time the sample was collected:** (i) on-arrival (sample time point 1), (ii) pen sampling (sample time point 2), or (iii) management of pen outbreak (sample time point 3).	**Accuracy** of laboratory results from each sampling time point **would be influenced by sample-level diagnostic sensitivity (Se) & specificity (Sp)** for detection of ARGs/mutations or phenotypic AMR for specific BRD pathogens.<br><br>**The Se & Sp of long read metagenomics would be influenced by**: (i) enrichment, DNA extraction & quality, host depletion, library preparation, (ii) flow cell performance, (iii) bioinformatics for pathogen & ARG detection (databases(s) & tool(s)), (v) whether AMR is encoded by genes or single point mutations, & (vi) the extent to which ARG detection predicts AMR phenotype.<br><br>**The Se & Sp of culture/AST are influenced by:** (i) sample quality &

Information Lane Kaizen: BRD information processes	Granularity	Frequency	Accuracy
	number of species and isolates tested for susceptibility, as well as the (ii) antimicrobials tested and the (iii) range of antimicrobial concentrations included in a laboratory test panel.		shipping time, (ii) the culture & species detection methods used, and (iii) the availability of evidence-based minimum inhibitory concentration (MIC) breakpoints for phenotypic interpretation.
**KAIZEN 2**<br><br>**PEN-LEVEL SUMMARY OF INDIVIDUAL LABORATORY, BRD TREATMENT, AND MORTALITY RECORDS**<br><br>**Pen-level laboratory, BRD treatment, and mortality summary** is an information process that was **identified as Kaizen,** as uploaded **laboratory test results (metagenomics or culture/AST data) could be summarized at the pen-level**.	Within the feedlot management software, (i) **individual calf laboratory test results** could be **compiled and summarized for each pen,** and (ii) the **95% confidence intervals (CIs) for the prevalence of reported laboratory outcomes** (ARGs/mutations or phenotypic AMR for specific BRD pathogens) could be **calculated. Outcomes could then be reported** for each of the available sample times: 1, 2 or 3.<br><br>The **95% CIs would vary** based on the **number of samples with data per pen.**<br><br>**Summarized pen-level laboratory results** would be	**As the pen-specific individual calf laboratory results become available** from each sampling time point, the feedlot management software could **automatically update** the **pen summary**. This **pen summary of the laboratory testing data with 95% CIs** for each sampled pen **would be available as data are received and uploaded in the feedlot software.** The **summarized laboratory results** in each pen report **would be reported with the time the sample was collected:** (i) on-arrival, (ii) pen sampling, or (iii) management of pen outbreak.<br><br>**Summarized pen-level laboratory results** could be	**Accuracy of the results** for each specific sampling time point **would be influenced by pen-level Se and Sp** for detection of ARGs/mutations or phenotypic AMR by BRD pathogen.<br><br>**Pen-level Se & Sp** would be influenced by: (i) **diagnostic Se & Sp of the assay for the individual samples,** (ii) **sample size at the pen level** (granularity), and the (iii) **user-defined pen-level AMR threshold** (prevalence or number of AMR positives).<br><br>**BRD pathogens are contagious,** spread between calves through nose-to-nose contact and respiratory aerosols. The **influence of sample size on accuracy of assessing pen**

**Information Lane Kaizen: BRD information processes**	**Granularity**	**Frequency**	**Accuracy**
Note: this pen-level summary of laboratory data could then be used in the Information Processing Lane to assess antimicrobial treatment strategies for BRD.	**compared against the pen-level user-defined AMR threshold**.<br><br>If the **user-defined AMR threshold is exceeded**, this information will be **flagged** in the pen summary and a **pen-specific alert notification will be** sent to the **veterinarian.**	**automatically compared against the pen-level user-defined AMR threshold.** If this AMR **threshold were exceeded at any of the sampling time points**, this information could **automatically trigger** a **pen-specific alert notification** to be sent to the **veterinarian.**	**AMR status** will depend on the **timing of sample collection relative to opportunities for transmission of BRD pathogens** among calves within a pen. **Pen-level prevalence of known BRD pathogens changes significantly throughout the early feeding period** (24,27).

Information Lane Kaizen: BRD information processes	Granularity	Frequency	Accuracy
**KAIZEN 3**<br><br>**FEEDLOT-LEVEL SUMMARY OF PEN-LEVEL LABORATORY, BRD TREATMENT, AND MORTALITY RECORDS**<br><br>**Feedlot-level laboratory, BRD treatment, and mortality summary** is an information process that was **identified as Kaizen** as **pen-level laboratory test results (metagenomics or culture/AST data) could be <u>summarized at the feedlot-level</u>**.<br><br>Note: this feedlot-level summary of laboratory data could then be used in the Information Processing Lane to assess antimicrobial treatment strategies for BRD.	Within the feedlot management software, (i) the **number and proportion of HR pens and/or outbreak pens sampled,** (ii) and the **prevalence of reported laboratory outcomes** (ARGs/mutations or phenotypic AMR for specific BRD pathogens) **with 95% CIs from each sampled pen** could be be **compiled and summarized. Feedlot-level summaries would be reported with 95% CIs adjusted for clustering by pen** for each available sample time: 1, 2 or 3.<br><br>This **feedlot-level report would illustrate** the **central tendency** and **uncertainty** in the **prevalence of ARGs/mutations or phenotypic AMR for specific BRD pathogens across sampled HR pens and/or outbreak pens** for each sample time.<br><br>The **number & identity** of **sampled pens where the user-defined AMR threshold had been**	**As laboratory results are uploaded**, the feedlot management software could **automatically update the sampled pen summary** for each sample time (1, 2 or 3) and then **automatically update** the **feedlot summary** for **all sampled pens within the feedlot**. The **summarized laboratory results** in the **feedlot report would be reported for each sample time points:** (i) on-arrival, (ii) pen sampling, or (iii) management of pen outbreak.<br><br>This **feedlot summary of laboratory results with 95% CIs adjusted for clustering by pen** would be **available as data are received and uploaded** to the feedlot management software.<br><br>The **number and identity** of **sampled pens where the user-defined AMR threshold had been exceeded (triggered pens)**	**Accuracy of the results for each specific sampling time point** would be influenced by **feedlot-level Se and Sp** for detection of ARGs or phenotypic AMR by BRD pathogen.<br><br>**Feedlot level Se and Sp** would be influenced by (i) the **individual sample diagnostic Se and Sp,** (ii) the **sample size at the pen level** (granularity), (iii) the **user defined pen-level AMR threshold** (prevalence or number of AMR positives), (iv) the **number of pens sampled**, (v) **central tendency** and **uncertainty in the prevalence of ARGs/mutations or phenotypic AMR for specific BRD pathogens across sampled HR pens,** and (vi) the **number of sampled pens that exceed the user-defined AMR threshold.**

**Information Lane Kaizen: BRD information processes**	**Granularity**	**Frequency**	**Accuracy**
	**exceeded (triggered pens)** would be **reported.**	would **automatically** be **updated.**	

Supplementary Table S3. The information quality matrix for the Kaizen (opportunities for continuous improvement) identified for the Information Processing Lane of the future state IQ-VSM (Figure 2, Supplementary Figure S3) of BRD treatment plans used in a western Canadian commercial beef cattle feedlot production system.

**Information Processing Lane Kaizen: BRD information assessment and processing**	**Granularity**	**Frequency**	**Accuracy**
KAIZEN 1<br><br>**BASED ON PEN-LEVEL LABORATORY RESULTS, WAS THE USER-DEFINED PEN-LEVEL AMR THRESHOLD EXCEEDED?**<br><br>This **information processing step** was **identified as Kaizen** as it provided an **opportunity** to **use** the **summarized pen-level laboratory test results (metagenomics or culture/AST data)** from each **triggered pen as part of the veterinarian's decision-making process**	As previously mentioned in the information lane, **if the user-defined pen-level AMR threshold had been exceeded at any of the sampling time points** in the current run, this information would **trigger** the feedlot management software to send a **pen-specific alert notification to the veterinarian.**<br><br>**The veterinarian** would **review** the **summarized laboratory results for the 'triggered pen', and determine if (i) the number of calves sampled, and (ii) the 95% CIs on the prevalence of reported laboratory outcomes** (ARGs/mutations or phenotypic AMR for specific BRD pathogens) **provide sufficient information to question the**	**To be most effective, pen specific laboratory test results** for **sample time point 1** and/or **sample time point 2 would be uploaded, summarized, and analyzed for the veterinarian to review prior to peak incidence for first treatment for BRD.** Typically, the veterinarian would be reviewing results for specific pens when alerted by the feedlot management software.<br><br>**The veterinarian could utilize the pen-level laboratory test results** to **inform decisions on the appropriateness of current BRD treatment protocols** for the (i) **sampled HR pen,** and +/- (ii) **other similar but non-sampled HR pens** during the current run**.**	The **accuracy** of decisions to adapt a treatment protocol for a specific 'triggered pen' would depend on the strength of the association between detected ARGs/mutations or AMR phenotype for specific BRD pathogens and response to BRD treatment.<br><br>The accuracy of any resulting decisions would also depend on the appropriateness of the pen-level, user-defined AMR threshold for informing expected treatment success and expectations for responsible AMU. Components that could influence the decision to adapt the treatment protocol for a pen might include: (i) by how much was the threshold exceeded? (ii) which BRD pathogen(s) were detected? (iii) which ARGs were

**Information Processing Lane Kaizen: BRD information assessment and processing**	**Granularity**	**Frequency**	**Accuracy**
regarding the **appropriateness of the current BRD treatment protocol** for each **triggered pen**.	**appropriateness of the current BRD treatment protocol for the 'triggered pen'** and potentially for **other similar but non-sampled neighboring pens** during the current run.	**Pen-specific laboratory test results** for **sample time point 3 (pen BRD outbreaks)** would be **uploaded, summarized, and analyzed for the veterinarian to review** in the event that similar non-sampled neighboring pens experience an outbreak of BRD.	detected? (iv) which sampling time point were data from? (v) what is the positive predictive value of the tests at the pen-level for resistance to the drug/pathogen? (vi) what are the economics of the potential protocol change?

**KAIZEN 2**<br><br>**DO CUMULATIVE FEEDLOT-LEVEL DATA ALIGN WITH CURRENT BRD TREATMENT DECISIONS?**<br><br>This **information processing step** was **identified as a Kaizen** as it provided an **opportunity** to **analyze and utilize** the **summarized feedlot-level laboratory results (metagenomics or culture/AST data) as part of the veterinarian's decision-making process** about the **appropriateness of the feedlot-level BRD treatment protocols in the current run** and potentially for subsequent runs.	The veterinarian could evaluate **feedlot-level laboratory results, i.e., detection of ARGs/mutations or AMR phenotype for specific BRD pathogens from sampled HR and outbreak pens** using feedlot management software.<br><br>The veterinarian could also consider the (i) **number of pens sampled,** (ii) **precision of AMR estimates at the pen level,** (iii) **number of triggered pens,** and (iv) **uncertainty of the AMR estimates at the feedlot-level** for subsequent decisions on **appropriateness of the current BRD treatment protocols at the feedlot-level, in addition to other information sources** such as (v) acute and chronic BRD morbidity rates, (vi) BRD mortality rate, (vii) gross post mortem results, and (viii) other diagnostic data**.**	The **<u>complete</u> AMR dataset for all sampled HR** and **outbreak pens** for each of sample times 1, 2 or 3 could be **automatically summarized** by the feedlot management software **as laboratory data become available.**<br><br>The **<u>complete</u> <u>summarized</u> AMR dataset for all sampled HR** and **outbreak pens should be available to the veterinarian in sufficient time** to **inform their decisions** regarding the **appropriateness of the feedlot-level BRD treatment protocols for the current run** and potentially for subsequent runs.	**Currently the veterinarian considers the** (i) acute and chronic BRD morbidity rates, (ii) BRD mortality, (iii) gross post-mortem results, (iv) other diagnostic data and (v) economics of a potential BRD protocol change **when questioning the appropriateness of the current feedlot-level BRD treatment protocols.**<br><br>The veterinarian could **also consider whether the <u>complete</u> <u>summarized</u> AMR dataset aligns with or suggests required changes to BRD treatment protocols in connection with the traditional morbidity and mortality dataset.**<br><br>The addition of AMR data from sampled HR and outbreak pens has the potential to **better inform the veterinarian when considering changes to BRD treatment protocols to achieve BRD treatment success** while considering antimicrobial stewardship goals for the industry.

References

24. Abi Younes, J.N.; Campbell, J.R.; Otto, S.J.G.; Gow, S.P.; Woolums, A.R.; Jelinski, M.; Lacoste, S.; Waldner, C.L. Variation in Pen-Level Prevalence of BRD Bacterial Pathogens and Antimicrobial Resistance Following Feedlot Arrival in Beef Calves. *Antibiotics (Basel)* 2024, 13, doi:10.3390/antibiotics13040322.

27. Abi Younes, J.N.; Campbell, J.R.; Gow, S.P.; Woolums, A.R.; Waldner, C.L. Association between respiratory disease pathogens in calves near feedlot arrival with treatment for bovine respiratory disease and subsequent antimicrobial resistance status. *Front Vet Sci* 2024, 11, 1-18, doi:10.3389/fvets.2024.1416436.
