# Supplementary material for: Opportunities for Laboratory Testing to Inform Antimicrobial Use for Bovine Respiratory Disease: Application of Information Quality Value Stream Maps in Commercial Feedlots
Source: Antibiotics (Basel). 2024 Sep 21;13(9):903. doi: 10.3390/antibiotics13090903 (PMC11428555; doi:10.3390/antibiotics13090903)

Future State Information Quality Value Stream Map for Bovine Respiratory Disease (BRD) Treatment Plan

Total production cycle time, western Canadian commercial feedlot scenario: 90 to > 300 days

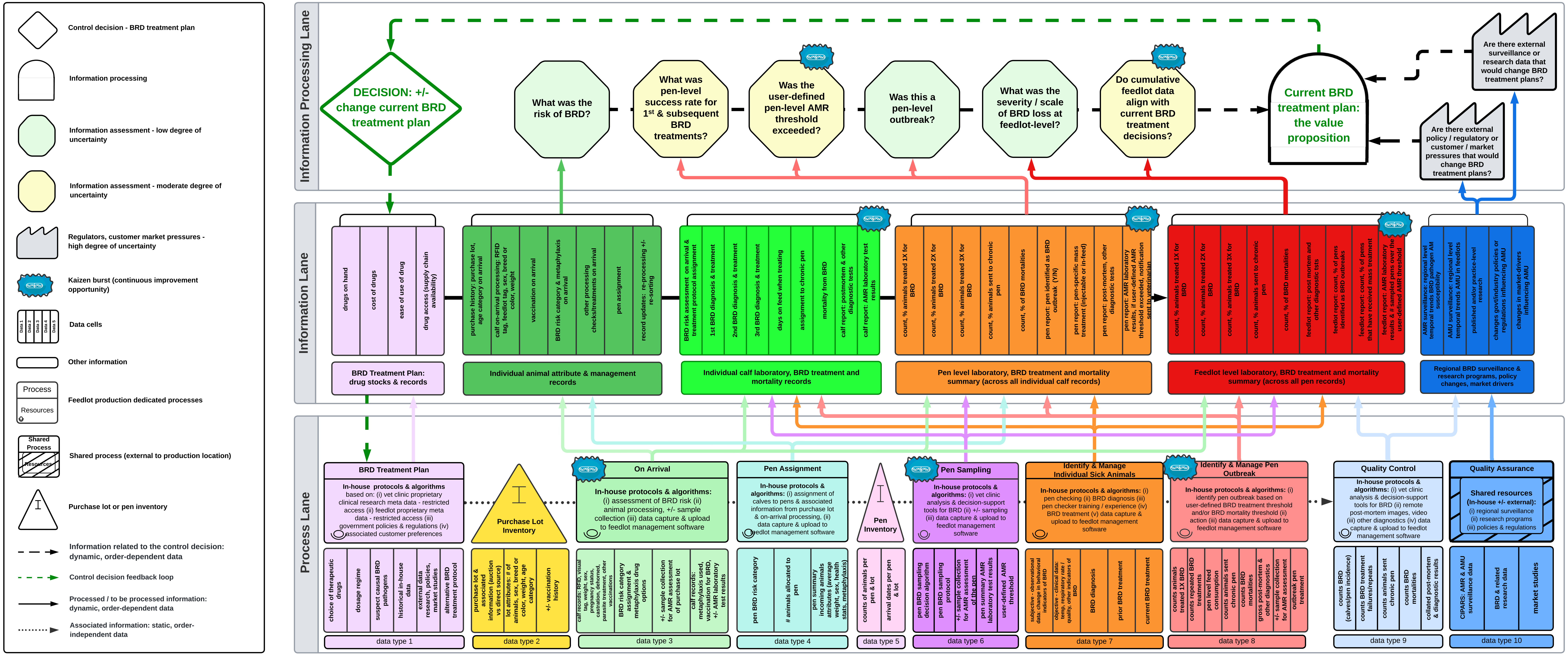

Supplement: Supplementary file 1 [file antibiotics-13-00903-s001.zip › SupplementaryMaterials4-FigureS3.pdf]
