# Supplementary figures and images for: Opportunities for Laboratory Testing to Inform Antimicrobial Use for Bovine Respiratory Disease: Application of Information Quality Value Stream Maps in Commercial Feedlots
Source: Antibiotics (Basel). 2024 Sep 21;13(9):903. doi: 10.3390/antibiotics13090903 (PMC11428555; doi:10.3390/antibiotics13090903)

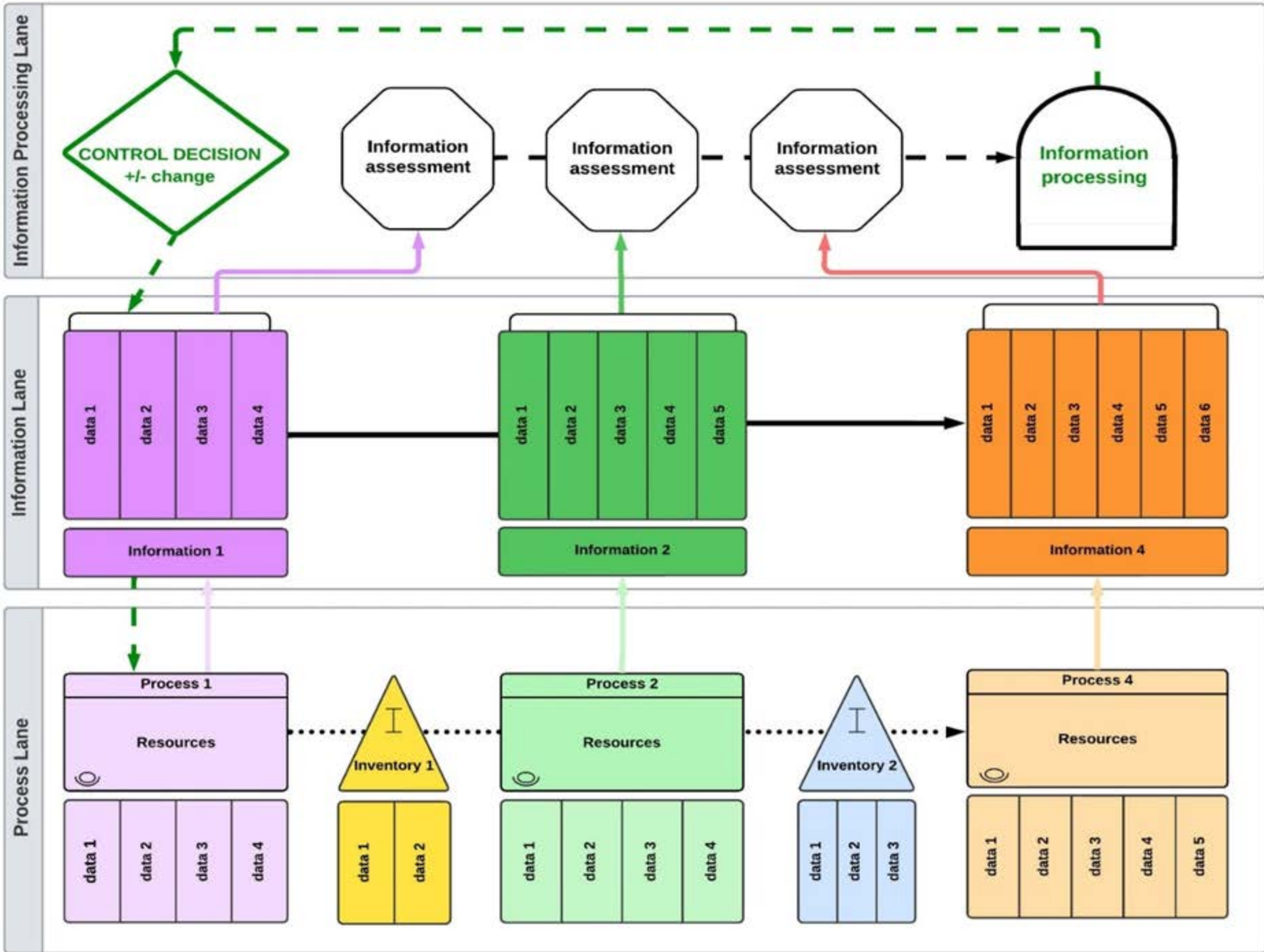

Supplement: Supplementary file 1 [file antibiotics-13-00903-s001.zip › SupplementaryMaterials2-FigureS1.pdf]
